# High MACC1 expression in combination with mutated KRAS G13 indicates poor survival of colorectal cancer patients

**DOI:** 10.1186/s12943-015-0316-2

**Published:** 2015-02-14

**Authors:** Katharina Ilm, Wolfgang Kemmner, Marc Osterland, Susen Burock, Gudrun Koch, Pia Herrmann, Peter M Schlag, Ulrike Stein

**Affiliations:** Experimental and Clinical Research Center, Charité University Medicine Berlin and Max-Delbrück-Center for Molecular Medicine, Robert-Rössle-Str.10, 13125 Berlin, Germany; Charité Comprehensive Cancer Center, Berlin, Germany; German Cancer Consortium, Heidelberg, Germany

**Keywords:** KRAS mutations, MACC1, Prognostic marker, Colorectal cancer, Metastasis-free survival

## Abstract

**Background:**

The metastasis-associated in colon cancer 1 (MACC1) gene has been identified as prognostic biomarker for colorectal cancer (CRC). Here, we aimed at the refinement of risk assessment by separate and combined survival analyses of MACC1 expression with any of the markers KRAS mutated in codon 12 (KRAS G12) or codon 13 (KRAS G13), BRAF V600 mutation and MSI status in a retrospective study of 99 CRC patients with tumors UICC staged I, II and III.

**Findings:**

We showed that only high MACC1 expression (HR: 6.09, 95% CI: 2.50-14.85, P < 0.001) and KRAS G13 mutation (HR: 5.19, 95% CI: 1.06-25.45, P = 0.042) were independent prognostic markers for shorter metastasis-free survival (MFS). Accordingly, Cox regression analysis revealed that patients with high MACC1 expression and KRAS G13 mutation exhibited the worst prognosis (HR: 14.48, 95% CI: 3.37-62.18, P < 0.001). Patients were classified based on their molecular characteristics into four clusters with significant differences in MFS (P = 0.003) by using the SPSS 2-step cluster function and Kaplan-Meier survival analysis.

**Conclusion:**

According to our results, patients with high MACC1 expression and mutated KRAS G13 exhibited the highest risk for metachronous metastases formation. Moreover, we demonstrated that the “Traditional pathway” with an intermediate risk for metastasis formation can be further subdivided by assessing MACC1 expression into a low and high risk group with regard to MFS prognosis. This is the first report showing that identification of CRC patients at high risk for metastasis is possible by assessing MACC1 expression in combination with KRAS G13 mutation.

**Electronic supplementary material:**

The online version of this article (doi:10.1186/s12943-015-0316-2) contains supplementary material, which is available to authorized users.

## Findings

### Background

Colorectal cancer (CRC) is the third most common cancer worldwide [1]. According to the state of the art for classification, colorectal carcinoma can be divided into three major pathways based on the underlying molecular alterations [2,3]. The majority of CRC belong to the “Traditional pathway” which reflects the adenoma-carcinoma sequence model [4,5]. Two additional pathways where recently described, the “Serrated” and “Alternate pathway” [2-4], which are characterized by differences in the microsatellite instability (MSI) status and mutations in the KRAS or BRAF oncogenes. In advanced stages metastatic spread shortens the 5-year survival rate from 90% to 10% [6,7]. Risk estimation by using genetic factors for early identification of high risk patients is still limited [8].

One promising candidate is the metastasis-associated in colon cancer 1 (MACC1) gene. MACC1 is an important prognostic marker for metastases formation in CRC [9,10].

Other promising molecular markers are oncogenic KRAS mutations, which are detectable in 30-50% of CRC tumors [11]. The most frequent point mutations in codon 12 (KRAS G12) and 13 (KRAS G13) of exon 2 result in constitutive activation of KRAS and downstream pathways [12]. Although the role of KRAS mutations as predictive biomarkers for anti-EGFR-targeted therapy is well characterized, there is a need to clarify their relevance as prognostic biomarkers for recurrence of metastatic CRC [13].

In this retrospective study, we attempted to improve the prognostic value of MACC1 for CRC metastasis using separate and combined survival analyses of MACC1 expression levels and KRAS G12 or G13 mutations in tumors of CRC patients. In addition, we investigated the prognostic significance of BRAF V600 mutation and MSI status, two of the most promising prognostic and predictive markers in the landscape of molecular biomarkers.

### Associations of MACC1 expression with the KRAS G12, KRAS G13 and BRAF V600 mutations as well as MSI status

In total, 99 patients with patho-histologically confirmed primary colorectal adenocarcinomas with Union for International Cancer Control (UICC) stages I, II and III were included in this study. This patient study protocol was approved by the Institutional Review Board (IRB) of the Charité Universitätsmedizin Berlin, complied with the Helsinki Declaration. The median metastasis-free survival (MFS) time and overall survival (OS) time of the patients were 56.0 and 79.4 months, respectively. This tumor marker study based on hypothesis-driven approaches was designed in compliance with the REMARK guidelines [14]. MACC1 mRNA expression level of the tumors were quantified using quantitative reverse transcriptase PCR [9]. All patient and tumor characteristics are shown in detail in the Additional file 1: Table S1–S4. For KRAS and BRAF mutation analyses as well as MSI status determination DNA sequencing methods were used [15,16]. All used material and methods are described in detail in Additional file 2. The molecular characteristics in this cohort (Additional file 1: Table S2) demonstrate the general distribution within colorectal tumors and mutual exclusivity of the oncogenes KRAS and BRAF [7,15].

**Figure 1:**
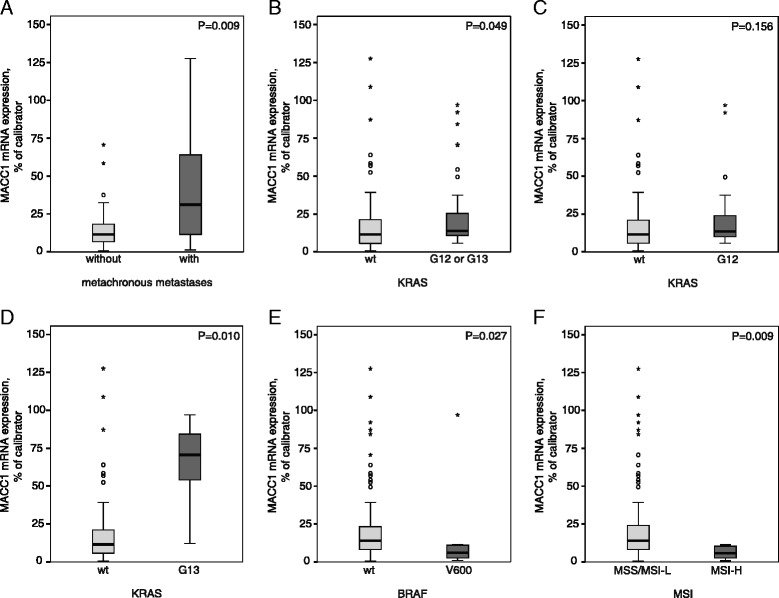
**Associations of MACC1 expression with KRAS mutation, BRAF mutation or MSI status.** MACC1 mRNA expression levels of the tumors were quantified using quantitative reverse transcriptase PCR [9]. Tumor DNA was extracted and subjected to PCR for amplification of KRAS and BRAF sequences and analyzed by sequencing [15]. MSI Analysis System was used for MSI status determination as described previously [16]. Tumors were discriminated in Microsatellite stable (MSS)/MSI-low (MSI-L; 0 or 1 markers demonstrating instability) or MSI-High (MSI-H; two or more markers instable). **A** Significantly increased MACC1 mRNA expression levels were detected in primary tumors of patients with metachronous metastases (P = 0.009). **B** MACC1 expression level was significantly increased in KRAS mutated (G12 or G13) tumors compared to tumors with KRAS wild type (KRAS wt) (P = 0.049). **C** There were no significant differences of MACC1 expression in KRAS G12 mutated compared to KRAS wt tumors (P = 0.156). **D** Primary tumors harboring G13 mutated KRAS showed a significantly increased MACC1 expression level (P = 0.010). **E** In BRAF wt tumors MACC1 expression was significantly increased compared to BRAF V600 mutated tumors (P = 0.027). **F** MACC1 expression of MSS/MSI-L tumors was significantly increased compared to MSI-H tumors (P = 0.009). Significance in differential mRNA expression was determined by Mann–Whitney *U*-test. Calculation of q-values from the corresponding P-values was performed to control for the false discovery rate (FDR) [17]. FDR-adjusted P-values less than 0.05 were considered to be statistically significant.

MACC1 expression was significantly higher in primary tumors of patients who developed metachronous metastases during the follow-up period (P = 0.009; Figure 1A), which confirmed previously described coherences [9,10]. We showed for the first time a correlation of high MACC1 expression with tumors harboring KRAS mutations (G12 or G13) (P = 0.049; Figure 1B) [7]. Separate analyses of primary tumors harboring the KRAS G13 mutation exhibited a significantly higher MACC1 expression (P = 0.010; Figure 1D), but not for KRAS G12 mutated tumors (P = 0.156; Figure 1C). High MACC1 expression also correlated with BRAF wild type (wt) (P = 0.027; Figure 1E) and microsatellite stable (MSS)/MSI-Low (MSI-L) (P = 0.009; Figure 1F) tumors.

### Association of MACC1 expression, KRAS G12 or G13 mutation, BRAF mutation and MSI status with MFS

**Figure 2:**
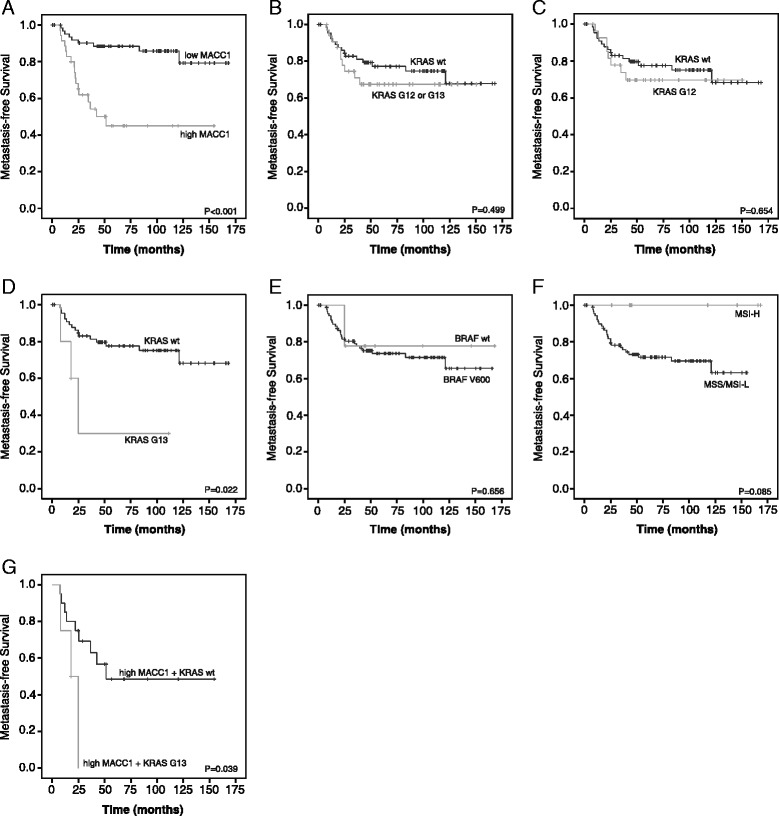
**Patients’ metastasis-free survival prognosis according to MACC1 mRNA expression, KRAS mutation, BRAF mutation and MSI status.** The Kaplan–Meier method was used to estimate cumulative survival rates. MFS time was defined as the time period from the date of surgery to the date of confirmed distant metastases or to the date of last follow-up contact/death for censored patients. **A** Patients with high MACC1 expression exhibited a statistically significant reduced MFS (P < 0.001). **B** No significant differences of MFS between KRAS mutated (G12 or G13) and KRAS wt tumors (P = 0.499) were detected. **C** KRAS G12 mutation showed no significant impact on MFS prognosis (P = 0.654). **D** MFS of patients with KRAS G13 mutated tumors compared to patients with KRAS wt was significantly reduced (P = 0.022). **E** There was no significant impact of BRAF V600 mutation on MFS prognosis (P = 0.656). **F** In MSS/MSI-L tumors we observed a tendency of shorter MFS (P = 0.085) compared to MSI-H tumors, but differences were not significant. **G** Tumors with high MACC1 expression and KRAS G13 exhibited the shortest MFS (mean: 19.0 months) compared to tumors with high MACC1 expression and KRAS wt (mean: 88.7 months, P = 0.039). Significance of differences in survival rates were assessed using the Log Rank test. P-values less than 0.05 were considered to be statistically significant.

We performed Kaplan-Meier survival analyses (Figure 2) and univariate Cox regression to estimate hazard ratios (HR) and 95% confidence intervals (CI) for the association of the tumor characteristics with MFS (Additional file 1: Table S5). Interestingly, univariate Cox regression and Log Rank test revealed that in the set of analyzed markers, only high MACC1 expression levels and KRAS G13 mutation emerged as prognostic factors for MFS. Patients with high MACC1 expression levels (Figure 2A, Log Rank P < 0.001; Cox regression: low vs. high, HR: 5.02, 95% CI: 2.19-11.53, P < 0.001) or KRAS G13 mutation (Figure 2D, Log Rank P = 0.022; Cox regression: wt vs. KRAS G13, HR: 4.25, 95% CI: 1.21-14.93, P = 0.024) in their primary tumors exhibited a significantly poorer prognosis. Although the role of KRAS G13 mutation as a predictive biomarker for anti-EGFR-targeted therapy [18] is well characterized, the prognostic value of KRAS G13 mutation in patients with CRC is controversial [19]. Various reports either encourage or dismiss KRAS mutations as prognostic biomarker for disease-specific survival rates or to predict liver or lung metastases [7,19-24]. Data of the Kirsten Ras in Colorectal Cancer Collaborative Group Studies (RASCAL I) suggested that the risk of recurrence and death is increased by the presence of KRAS mutations [11], and the updated RASCAL II study confined the correlation of KRAS mutation and survival prognosis to the G12V mutation [25]. According to our results, comparison of mutated KRAS tumors with KRAS wt tumors had no significant impact on MFS (Figure 2B, Log Rank P = 0.499; Cox regression: wt vs. KRAS G12 or G13, HR: 1.40, 95% CI: 0.63-3.09, P = 0.409). Studies comparing the effects of KRAS G12 with KRAS G13 mutations are rare [19,24]. Therefore separate analyses of KRAS G12 and KRAS G13 mutations in the tumors were included and demonstrated that especially the KRAS G13, but not KRAS G12 (Figure 2C, Log Rank P = 0.654; Cox regression: wt vs. KRAS G12, HR: 1.29, 95% CI: 0.55-3.03, P = 0.556), mutations were prognostic for reduced MFS. Separate analyses of the most prominent G12 mutations (G12V, G12D and G12C) revealed no significant impact of these mutations on MFS prognosis (Log Rank P = 0.826, P = 0.896 and P = 0.766; respectively). Furthermore, BRAF V600 mutation (Figure 2E, Log Rank P = 0.656; Cox regression: wt vs. BRAF V600, HR: 0.75, 95% CI: 0.18-3.17, P = 0.694) or MSS/MSI-L (Figure 2F, Log Rank P = 0.085; Cox regression: MSS/MSI-L vs. MSI-H, HR: 0.04, 95% CI: 0.00-12.05, P = 0.272) were also not associated with worse prognosis.

### Combinatorial survival analyses of MACC1 expression and KRAS G12 or G13 mutation with tumor characteristics

Next, we analyzed the effects of clinicopathological parameters like age, gender, UICC stage, grading and postoperative treatment of the tumors concerning MFS in combination with MACC1 or KRAS mutations using multivariate Cox regression (Table 1). Only high MACC1 expression (HR: 6.09, 95% CI: 2.50-14.85, P < 0.001) and KRAS G13 mutation (HR: 5.19, 95% CI: 1.06-25.45, P = 0.042) turned out to be independent prognostic markers for shorter MFS in the set of analyzed biomarkers. To our knowledge, this is the first study showing a prognostic relevance of KRAS G13 for MFS in CRC patients and demonstrating KRAS G13 mutation as independent risk factor of age, gender, UICC stage, grading and postoperative treatment for metachronous metastases development.

**Table 1:** **Impact of clinicopathological parameters in combination with MACC1 and KRAS mutation status concerning MFS**

**Covariates**	**Multivariate MACC1**		**Multivariate mutated (G12 or G13) KRAS**		**Multivariate G13 mutated KRAS**		**Multivariate G12 mutated KRAS**	
	**P-value**	**HR (95%** **CI)**	**P-value**	**HR (95%** **CI)**	**P-value**	**HR (95%** **CI)**	**P-value**	**HR (95%** **CI)**
**Molecular Markers MACC1 (low vs. high) KRAS G12/G13 (wt vs. mutated)**	<0.001	6.09 (2.50-14.85)	0.302	1.55 (0.67-3.59)	0.042	5.19 (1.06-25.45)	0.543	1.34 (0.52-3.42)
**Age at diagnosis (<60) vs. (>60) years**	0.970	1.02 (0.41-2.52)	0.892	0.94 (0.39-2.26)	0.648	1.30 (0.43-3.93)	0.940	1.04 (0.37-2.90)
**By gender Male vs. Female**	0.890	0.94 (0.41-2.17)	0.969	1.02 (0.44-2.34)	0.494	1.42 (0.52-3.91)	0.963	0.98 (0.41-2.35)
**UICC stage (I) vs. (II + III)**	0.473	1.65 (0.42-6.53)	0.191	2.42 (0.64-9.09)	0.356	2.16 (0.42-11.10)	0.115	3.59 (0.73-17.54)
**By grading (G1 + 2) vs. (G3 + 4)**	0.416	0.64 (0.22-1.88)	0.257	0.53 (0.18-1.59)	0.305	0.45 (0.10-2.06)	0.094	0.28 (0.06-1.24)
**By postoperative treatment Untreated vs. treated**	0.064	2.37 (0.95-5.93)	0.447	1.40 (0.59-3.29)	0.723	1.22 (0.41-3.57)	0.483	1.39 (0.55-3.49)

The P-values, hazard ratios (HR) and 95% confidence intervals (CI) of different parameters concerning MFS were calculated using Cox regression analyses. The analysis was performed separately for each factor with the parameters listed in the table being introduced as covariates, respectively.

In addition, we performed multivariate Cox regression combining MACC1 expression level with the tumor characteristics KRAS G12 or G13 mutation, BRAF V600 mutation and MSI status (Additional file 1: Table S5). This analysis revealed that MACC1 was statistically significant independent for MFS prognosis of these molecular markers (HR: 4.20, 95% CI: 1.80-9.81, P = 0.001). Therefore, we conducted combinatorial survival prognosis of the independent prognostic markers MACC1 expression and KRAS G13 mutation. Interestingly, patients with the worst prognosis were characterized by high MACC1 expression and KRAS G13 mutation (HR: 14.48, 95% CI: 3.37-62.18, P < 0.001). These patients had the shortest MFS time (Figure 2G, Log Rank: P = 0.039 mean: 18.99 months, 95% CI: 10.64-27.34) compared to patients with high MACC1 expression and KRAS wt tumors (Additional file 1: Table S6). The identification of CRC patients at high risk for metastasis formation is possible by assessing MACC1 expression in combination with KRAS G13 mutations.

### Cluster classification

**Figure 3:**
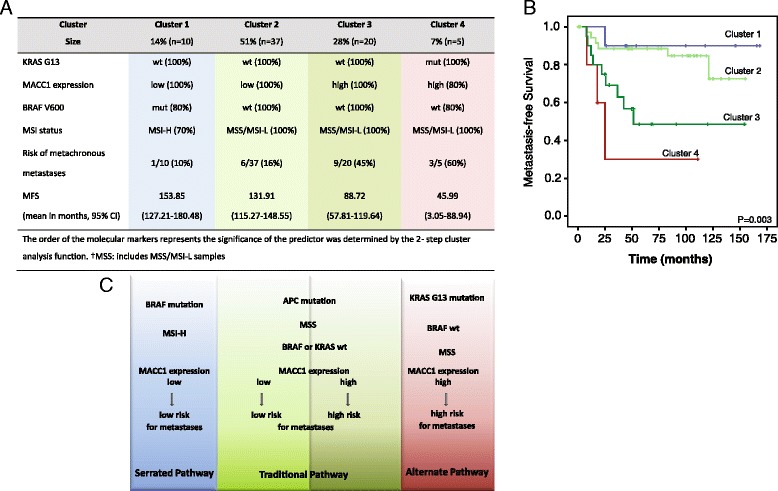
**Classification of CRC patients with regard to their molecular characterization. A** Integrative 2-step cluster analyses allowed the differentiation of four clusters with regard to their tumor characteristics. Clustering of the tumors concerning KRAS G13 mutation, MACC1 expression, BRAF V600 mutation and MSI status was performed by the SPSS 2-step cluster function. The order of the molecular markers represents the significance of the predictor was determined by the 2- step cluster analysis function. †MSS: includes MSS/MSI-L samples. **B** Kaplan-Meier survival analysis revealed significantly different survival times for the four clusters (P = 0.003). Patients of Cluster 1 had the best prognosis and patients in Cluster 4 had the worst prognosis. **C** According to Leggett and Whitehall [2] Cluster 1 (in blue) represents the “Serrated pathway” which is characterized by the lowest risk (10%) for metastasis formation. Cluster 2 and Cluster 3 belong to the “Traditional pathway” (in green) with intermediate risk for metastasis formation. Here, we show that MACC1 expression subdivides this cluster into a low risk (16%, light green) and high risk (45%, dark green) group. The highest risk (60%) patients were classified in Cluster 4 representing the “Alternate pathway” (in red). According to our results these tumors are now characterized by a high MACC1 expression and mutated KRAS G13.

Using integrative 2-step cluster analysis based on the presence of the proven markers in our cohort four clusters of patients with a different risk for metastasis formation could be distinguished and coincide well with the presumed pathways (Figure 3). According to CRC classification by Leggett and Whitehall [2], Cluster 1 represents the “Serrated pathway” with MSI-H, BRAF mutation and low MACC1 expression. These patients showed the lowest risk for metachronous metastases formation. In our study Cluster 2 and 3 represents the “Traditional pathway”, where the majority of CRC cases belong to. This pathway is characterized by MSS and KRAS or BRAF mutation. Cluster 2 with a low risk for metastasis formation was further characterized by low MACC1 expression. In contrast, Cluster 3 with increased risk exhibited high MACC1 expression levels. Thus, MACC1 expression allows a further subdivision of the “Traditional pathway” into a low and high risk group of patients concerning metastasis formation. Patients in Cluster 4 had the highest risk for metachronous metastases development. These tumors belonging to the “Alternate pathway” were defined by MSI-L/MSS, KRAS G13 mutation and can be further characterized by high MACC1 expression.

## Conclusion

This exploratory study has limitations especially due to the limited number of patient samples. But this cohort has also several strengths: The marker analyses were performed with microdissected cryosections verified by an experienced pathologist. Furthermore the studied cohort is very well characterized and only samples from preoperatively untreated patients with very long follow-up data and complete clinicopathological information were used.

In conclusion, our results underline the high relevance of MACC1 and KRAS G13 mutation for CRC metastasis prognosis. This is the first study showing a prognostic relevance of KRAS G13 for MFS in CRC patients. Furthermore, high MACC1 expression and KRAS G13 mutation are independent prognostic markers for metachronous metastases development. Accordingly, combinatorial survival prognosis revealed that patients with high MACC1 expression and KRAS G13 mutation exhibited the worst prognosis.

Interestingly, the conventional CRC classification can be extended and refined by inclusion of MACC1 expression and mutated KRAS G13 as independent markers. Therefore, this refinement allows improved individualized prognosis and intervention strategies for the majority of CRC patients in clinical routine.

## Consent

Written informed consent was obtained from the patient for the publication of this report and any accompanying images.

## Additional files

Additional file 1:
**Supplementary Tables.** **Table S1.** Patient characteristics with regard to MACC1 expression and the determined relative risk (RR) with corresponding 95% confidence intervals (CI) are shown. **Table S2.** The frequencies of mutations and MSI status of the tumors and corresponding MACC1 expression are shown. **Table S3.** Contains the localization of the metachronous, distant metastases of the patients within the cohort. **Table S4.** The frequencies of postoperative treatment within the patient cohort are shown. **Table S5.** Includes the univariate and multivariate Cox regression analyses of the analyzed biomarkers. The analysis was performed separately for each factor with the parameters listed in the table being introduced as covariates, respectively. **Table S6.** Includes the MFS time and P-values for the analyzed biomarkers were calculated using the Kaplan–Meier survival analyses and the Log Rank test. Additional file 2:
**Materials and methods.** This file includes detailed information regarding tumor specimens, RNA and DNA isolation, mutations analysis, MSI status determination and statistical analysis.

## Abbreviations

BRAF: v-Raf murine sarcoma viral oncogene homolog B
CI: Confidence interval
CRC: Colorectal cancer
EGFR: Epidermal growth factor receptor
HR: Hazard ratio
FDR: False discovery rate
KRAS: Kirsten rat sarcoma viral oncogene homolog
MACC1: Metastasis-associated in colon cancer 1
MFS: Metastasis-free survival
MSI (−L/H): Microsatellite instability (−low/high)
MSS: Microsatellite stable
RASCAL: Kirsten Ras in Colorectal Cancer Collaborative Group
RR: Relative risk
UICC: Union for International Cancer Control
wt: Wild type
