# Supplementary material for: High MACC1 expression in combination with mutated KRAS G13 indicates poor survival of colorectal cancer patients
Source: Mol Cancer. 2015 Feb 14;14:38. doi: 10.1186/s12943-015-0316-2 (PMC4335361; doi:10.1186/s12943-015-0316-2)
Supplement: Additional file 1: — Supplementary Tables. Table S1. Patient characteristics with regard to MACC1 expression and the determined relative risk (RR) with corresponding 95% confidence intervals (CI) are shown. Table S2. The frequencies of mutations and MSI status of the tumors and corresponding MACC1 expression are shown. Table S3. Contains the localization of the metachronous, distant metastases of the patients within the cohort. Table S4. The frequencies of postoperative treatment within the patient cohort are shown. Table S5. Includes the univariate and multivariate Cox regression analyses of the analyzed biomarkers. The analysis was performed separately for each factor with the parameters listed in the table being introduced as covariates, respectively. Table S6. Includes the MFS time and P-values for the analyzed biomarkers were calculated using the Kaplan–Meier survival analyses and the Log Rank test. [file 12943_2015_316_MOESM1_ESM.docx]

**Additional file 1 (Title of data: Tables)**

**Ilm et al: High MACC1 expression in combination with mutated KRAS G13 indicates poor survival of colorectal cancer patients**

**Table S1: Patient characteristics with regard to MACC1 expression**

| **Criteria** | **MACC1 expression** | | **P-value** | **RR (95% CI)** |
| --- | --- | --- | --- | --- |
|  | **low (n=64)** | **high (n=35)** |  |  |
| **Gender** |  |  | **0.952** | 0.99 (0.74-1.332) |
| **Male** (n=59) | 38 (59.38%) | 21 (60.00%) |  |  |
| **Female** (n=40) | 26 (40.63%) | 14 (40.00%) |  |  |
| **Age at diagnosis** |  |  | **0.797** | 1.04 (0.76-1.44) |
| **<60 years** (n=27) | 18 (28.13%) | 9 (25.71%) |  |  |
| **≥60 years** (n=72) | 46 (71.88%) | 26 (74.29%) |  |  |
| **Median** $\boldsymbol{\pm}$ **SD:**  **64.8 years** $\boldsymbol{\pm}$ **9.6** | **64.3 years** $\boldsymbol{\pm}$ **9.5** | **65.3 years** $\boldsymbol{\pm}$ **10.2** |  |  |
| **UICC stage** |  |  | **0.369** | 1.10 (0.80-1.52) |
| **UICC I+II** (n=66) | 44 (68.75%) | 22 (62.86%) |  |  |
| **UICC III** (n=33) | 20 (31.25%) | 13 (37.14%) |  |  |
| **pT status** |  |  | **0.569** | 1.10 (0.81-1.50) |
| **pT1+2** (n=26) | 18 (28.13%) | 8 (22.86%) |  |  |
| **pT3+4** (n=73) | 46 (71.88%) | 27 (77.14%) |  |  |
| **pN status** |  |  | **0.552** | 1.10 (0.80-1.52) |
| **Negative** (n=66) | 44 (68.75%) | 22 (62.88%) |  |  |
| **Positive** (n=33) | 20 (31.25%) | 13 (37.14%) |  |  |
| **Grading (G)** |  |  | **0.827** | 0.96 (0.68-1.36) |
| **G1+G2** (n=78) | 50 (78.13%) | 28 (80.00%) |  |  |
| **G3+G4** (n=21) | 14 (21.88%) | 7 (20.00%) |  |  |
| **Localization^#^** |  |  | **0.133** | 1.33 (0.90-1.98) |
| **Colon** (n=71) | 49 (76.56%) | 22 (62.86%) |  |  |
| **Rectum** (n=27) | 14 (21.88%) | 13 (37.14%) |  |  |
| **Metachronous Metastases** | |  | **<0.001***** | 2.18 (1.26-3.75) |
| **Without** (n=73) | 55 (85.94%) | 18 (51.43%) |  |  |
| **With** (n=26) | 9 (14.06%) | 17 (48.57%) |  |  |
| Tumors were classified according to the guidelines of the Union for International Cancer Control (UICC) staging system. Receiver operating characteristic (ROC) analysis was used to denominate low and high MACC1 mRNA expression groups. In the resulting ROC curve the calculated AUC was 0.699. Based on the coordinates for sensitivity and 1-specificity the optimal ROC cutoff for the MACC1 expression was 226.81. The chi-square ($\boldsymbol{\chi}^{\boldsymbol{2}}$) test was used to compare MACC1 expression with clinicopathological factors. The relative risk (RR) with corresponding 95% confidence intervals (CI) was calculated.  ^#^One unknown case (1.56%) was excluded for analysis.  SD: standard deviation, pT: post-operative tumor stage, pN: post-operative lymph node status | | | | |

**Table S2:** **Frequencies of mutations and MSI status of the tumors and corresponding MACC1 expression**

| Criteria | MACC1 expression | |
| --- | --- | --- |
|  | **low (n=64)** | **high (n=35)** |
| KRAS mutation |  |  |
| Wild type (n=66) | 46 (71.88%) | 20 (57.14%) |
| G12 mutation (n=28) | 17 (26.56%) | 11 (31.43%) |
| G12V (n=12) | 11 (17.19%) | 1 (2.86%) |
| G12D (n=9) | 3 (4.69%) | 6 (17.14%) |
| G12C (n=4) | 1 (1.56%) | 3 (8.57%) |
| G12A (n=1) | 1 (1.56%) | 0 (0.00%) |
| G12S (n=1) | 1 (1.56%) | 0 (0.00%) |
| G12G (n=1) | 0 (0.00%) | 1 (2.86%) |
| G13 mutation (n=5) | 1 (1.56%) | 4 (11.43%) |
| MSI status |  |  |
| MSS/MSI-L (n=91) | 56 (87.50%) | 35 (100.00%) |
| MSI-H (n=8) | 8 (12.50%) | 0 (0.00%) |
| BRAF mutation |  |  |
| Wild typ (n=90) | 56 (87.50%) | 34 (97.14%) |
| Mutated (n=9) | 8 (12.50%) | 1 (2.86%) |

**Table S3: Localization of the metachronous, distant metastases**

| Localization of metastases | MACC1 expression | |
| --- | --- | --- |
|  | **low (n=8)** | **high (n=17)** |
| Liver (n=12) | 4 (50.00%) | 8 (47.06%) |
| Lung (n=5) | 1 (12.50%) | 4 (23.53%) |
| Peritoneum (n=2) | 0 (0.00%) | 2 (11.76%) |
| Liver and Lung (n=6) | 3 (37.50%) | 3 (17.65%) |

**Table S4: Postoperative treatment of patients**

| Postoperative treatment | MACC1 expression | |
| --- | --- | --- |
|  | **low (n=64)^#^** | **high (n=35)** |
| Untreated (n=61) | 41 (64.06%) | 20 (57.14%) |
| Chemotherapy (n=30) | 18 (28.13%) | 12 (34.29%) |
| Radiotherapy (n=1) | 0 (0.00%) | 1 (2.86%) |
| Combination (Radio- and Chemotherapy) (n=5) | 3 (4.69%) | 2 (5.71%) |
| ^#^includes two patients (3.13%) without information about adjuvant therapy | | |

**Table S5: Analyzing the association of tumor characteristics alone and in combination with regard to MFS**

|  | Univariate | | Multivariate | |
| --- | --- | --- | --- | --- |
|  | **P-value** | **HR (95% CI)** | **P-value** | **HR (95% CI)** |
| MACC1 expression | <0.001 | 5.02 (2.19-11.53) | 0.001 | 4.20 (1.80-9.81) |
| KRAS G12 | 0.556 | 1.29 (0.55-3.03) | 0.814 | 0.90 (0.38-2.14) |
| KRAS G13 | 0.024 | 4.25 (1.21-14.93) | 0.224 | 2.48 (0.58-10.45) |
| BRAF V600 | 0.694 | 0.75 (0.18-3.17) | 0.644 | 1.51 (0.27-8.53) |
| MSI | 0.272 | 0.04 (0.00-12.05) | 0.981 | 0 (0-0) |
| The P-values, hazard ratios (HR) and 95% confidence intervals (CI) of different parameters concerning MFS were calculated using Cox regression analyses. | | | | |

**Table S6: Results of MFS prognoses**

| **Criteria** | **Status** | **MFS**  **(mean in months, 95% CI)** | **P-value** |
| --- | --- | --- | --- |
| **MACC1 expression** | **low** | 145.61 (132.02-159.19) | **<0.001** |
|  | **high** | 83.02 (59.32-106.72) |  |
| **KRAS wild type vs. KRAS G12**  **or KRAS G13 mutation** | **wt** | 127.65 (111.15-144.15) | **0.499** |
|  | **KRAS G12 or KRAS G13** | 108.47 (87.03-129.91) |  |
| **KRAS wild type vs.**  **KRAS G12 mutation** | **wt** | 130.17 (114.05-146.29) | **0.555** |
|  | **KRAS G12** | 111.85 (89.49-134.21) |  |
| **KRAS wild type vs.**  **KRAS G13 mutation** | **wt** | 130.17 (114.05-146.29) | **0.014** |
|  | **KRAS G13** | 45.99 (3.05-88.94) |  |
| **KRAS wt**  **+ MACC1 expression** | **low** | 145.27 (131.50-159.03) | **<0.001** |
|  | **high** | 88.83 (63.93-113.73) |  |
| **High MACC1 expression**  **+ KRAS mutation status** | **wt** | 88.72 (57.81-119.64) | **0.039** |
|  | **KRAS G13** | 18.99 (10.64-27.34) |  |
| The Kaplan–Meier method was used to estimate cumulative survival rates and MFS time. Significance of differences in survival rates were assessed using the Log Rank test. | | | |
