# Supplementary material for: High MACC1 expression in combination with mutated KRAS G13 indicates poor survival of colorectal cancer patients
Source: Mol Cancer. 2015 Feb 14;14:38. doi: 10.1186/s12943-015-0316-2 (PMC4335361; doi:10.1186/s12943-015-0316-2)
Supplement: Additional file 2: — Materials and methods. This file includes detailed information regarding tumor specimens, RNA and DNA isolation, mutations analysis, MSI status determination and statistical analysis. [file 12943_2015_316_MOESM2_ESM.docx]

**Additional file 1 (Title of data: Material and Methods)**

**Ilm et al: High MACC1 expression in combination with mutated KRAS G13 indicates poor survival of colorectal cancer patients**

**Patient characteristics**

In total, 99 patients with patho-histologically confirmed primary colorectal adenocarcinomas with UICC stages I, II and III were included in this study (Additional file 2, Supplementary Table 1). The median for metastasis-free survival time (MFS) and overall survival (OS) time was 56.0 and 79.4 months, respectively. Primary tumors were obtained from all patients with informed written consent and collected between 1996 and 2003. This patient study protocol was approved by the Institutional Review Board (IRB) of the Charité Universitätsmedizin Berlin, complied with the Helsinki Declaration. Tumor staging and typing was performed according to UICC and WHO guidelines. The patients were preoperatively untreated, had no history of familial CRC, did not suffer from a second tumor and underwent R0 resection. Primary tumor tissues were collected after surgical removal and were immediately shock frozen in liquid nitrogen after surgery. Cryoconserved tumor specimens were obtained from the tumor bank of the Charité Comprehensive Cancer Center (Berlin). Cryosections of all samples were performed and every fifth section was stained with hematoxylin. Histopathology of each sample used for experimental analysis was reviewed by an experienced pathologist to confirm diagnosis, tissue composition and tumor content. After cryosectioning of CRC samples we applied microdissection of unstained slides to obtain almost pure tumor cell populations for DNA and RNA isolation. This study was designed and executed in compliance with the Reporting Recommendations for Tumor Marker Prognostic Studies (REMARK) guidelines [14].

**DNA and RNA isolation**

Genomic DNA was isolated from cryoconserved tumor tissues using Charge Switch gDNA Micro Tissue Kit (Invitrogen) according to the manufacturer’s protocol. Total RNA was isolated using TRIzol reagent (Invitrogen), including a DNase step. Eluted DNA and RNA were quantified using Nanodrop 1000 (Peqlab Biotechnologie). RNA quality was proven using 2100 Bioanalyzer (Agilent).

**KRAS and BRAF mutation analyses**

The extracted DNA was subjected to PCR and KRAS (forward primer, 5’-GGT GGA GTA TTT GAT AGT GTA TTA ACC-3’; reverse primer, 5’-AGA ATG GTC CTG CAC CAG TAA-3’) and BRAF (forward primer, 5’-CCTAAACTCTTCATAATGCTTGCTC-3’; reverse primer, 5’-CCACAAAATGGATCCAGACA-3’) sequences were amplified. The PCR conditions were as follows: 95°C for 5 minutes, 40 cycles of 95°C for 30 seconds, 61°C for 30 seconds, and 72°C for 40 seconds, 72°C for 5 minutes [15]. PCR products were purified using QIAquick®PCR Purification Kit (Qiagen) according to the manufacturer’s instruction and sent to Stratec (Berlin) sequencing service for sequencing. As positive controls for KRAS, DNAs from the cell lines SW480 (KRAS G12 mutation) and HCT116 (KRAS G13 mutation) were included. DNA from the cell line Colo205 was included as positive control for BRAF V600 mutation.

**Microsatellite analysis (MSI status)**

DNA was analyzed using the MSI Analysis System (Promega, Madison, USA) according to the manufacturer’s protocol, including five mononucleotides (NR-21, BAT-26, BAT-25, NR-24, MONO-27; genes SLC7A8, MSH2, c-kit, ZNF-2 and MAP4K3, respectively). These mononucleotides are the most sensitive and specific markers for detection of MSI-H tumors. In addition, two pentanucleotide markers were included for identification of sample cross contaminations [16]. PCR products were sequenced on an ABI 3700 sequencer (Applied Biosystems). Tumors were discriminated in Microsatellite stable (MSS)/MSI-low (MSI-L) (0 or 1 markers demonstrating instability) or MSI-H (two or more markers instable).

**Quantitative real-time RT-PCR (qRT-PCR)**

Reverse transcriptase reaction was performed with 50 ng of total RNA. Quantitative real-time PCR (95°C 60 seconds, 45 cycles of 95°C 10 seconds, 60°C 10 seconds, 72°C 20 seconds) was performed for quantification of the MACC1 expression (forward primer, 5’- TTC TTT TGA TTC CTC CGG TGA -3‘; reverse primer, 5‘-ACT CTG ATG GGC ATG TGC TG-3’; FITC probe, 5’-GCA GAC TTC CTC AAG AAA TTC TGG AAG ATC TA-3’; LCRed 640 probe, 5’- AGT GTT TCA GAA CTT CTG GAC ATT TTA GAC GA-3’; BioTeZ and TIB MolBiol) using the LightCycler480 (DNA Master Hybridization Probes kit, Roche Diagnostics) as previously described [9].

**Statistics**

All calculations and statistical analyses were performed with IBM® SPSS® Statistics 21. Receiver operating characteristics (ROC) analysis was performed on MACC1 mRNA expression levels to denominate low and high MACC1 expression groups for further analyses. In the resulting ROC curve the calculated AUC was 0.699. Based on the coordinates for sensitivity and 1-specificity the optimal ROC cutoff for the MACC1 expression was 226.81. For retrospective analyses clinical follow-up information was obtained from the tumor bank of the Charité Comprehensive Cancer Center (Berlin) after completed mutation analyses and mRNA quantification. The chi-square ($\chi^{2}$) test was used to compare MACC1 expression with clinicopathological factors. Furthermore, the relative risk (RR) with corresponding 95% confidence intervals (CI) was calculated (Additional file 2, Supplementary Table 1). Distribution of MACC1 mRNA expression levels with regard to the different parameters were examined using box and whisker plots. Significance in differential mRNA expression was determined by Mann-Whitney U-test. Calculation of *q*-values from the corresponding *p-*values was performed to control for the false discovery rate [17]. The *q*-values for this study were calculated using QVALUE software from the non-corrected *p*-values. FDR-adjusted *p-*values less than 0.05 were considered to be statistically significant.

The Kaplan–Meier method was used to estimate cumulative survival rates and significance of differences in survival rates were assessed using the Log Rank test. MFS time was defined as the time period from the date of surgery to the date of confirmed distant metastases or to the date of last follow-up contact/death for censored patients. The p-values, hazard ratios (HR) and 95% CI of different parameters concerning MFS were calculated using univariate und multivariate Cox regression. The analysis was performed separately for each factor with the parameters listed in Table 1 and in Additional file 2, Supplementary Table 5 being introduced as covariates, respectively. Clustering of the tumors concerning KRAS G13 mutation, MACC1 expression, BRAF V600 mutation and MSI status was performed by the SPSS 2-step cluster function (Fig. 3). Tumors harboring mutations in codon 12 (KRAS G12) were excluded for the 2-step cluster analysis.
